# Effect of Temperature on Ultrasonic Nonlinear Parameters of Carbonated Concrete

**DOI:** 10.3390/ma15248797

**Published:** 2022-12-09

**Authors:** Jinzhong Zhao, Jin Wu, Xuejun Chen, Ruifu Zeng

**Affiliations:** 1 College of Civil Aviation, Nanjing University of Aeronautics and Astronautics, Nanjing 210016, China; 2 Eastern Airports, Nanjing 210016, China

**Keywords:** concrete carbonation, nonlinear parameter, temperature effects, embedded composite piezoelectric transducer, nondestructive testing

## Abstract

In order to explore the monitoring technique of concrete carbonation in various temperatures, longitudinal ultrasonic nonlinear parameters of carbonated concrete are measured by using an embedded composite piezoelectric transducer (ECPT) and a surface-mounted transducer. The effect of temperature from −20 ${}^{\circ }$C to 40 ${}^{\circ }$C with a temperature interval of 5 ${}^{\circ }$C and water–cement ratio on the measurements of ultrasonic parameters for carbonated concrete is investigated. The ultrasonic transmission detection method and the second harmonic generation (SHG) technique for longitudinal waves are used in the study. Results of the experiment demonstrate that ECPT is effective in the monitoring of the changes in ultrasonic parameters of carbonated concrete. At the temperature ranging from 15 ${}^{\circ }$C to 40 ${}^{\circ }$C, the increasing temperature slightly increases the relative nonlinear parameters of carbonated concrete. It decreases significantly that the relative nonlinear parameters of carbonated concrete measured at 0 ${}^{\circ }$C compared with that at 10 ${}^{\circ }$C. The configuration in this measurement is also appropriate for the assessment of carbonated concrete during carbonation time in low-temperature environments (below 0 ${}^{\circ }$C). In the same carbonation time, the relative nonlinear parameters also increase slightly when the temperature is at −20 ${}^{\circ }$C to 0 ${}^{\circ }$C, but it does not change too much. Furthermore, there is a more significant variation of the nonlinear parameters in the same carbonation time for the specimens with a high water–cement ratio than that with a low one.

## 1. Introduction

Ultrasonic measurement as non-destructive testing technology has been widely used to determine the characterization of concrete structures for a long time. Nonlinear resonance ultrasonic spectroscopy (NRUS) technology has been used in the testing of progressive damage occurring in concrete, such as alkali–silica reaction [1], thermal damage [2], and delayed ettringite formation in concrete [3]. These progressive changes in concrete increase the material nonlinearity, resulting in a shift of the resonance frequency and amplitude in concrete. During concrete carbonation, the material nonlinearity is also changed by the effects of the carbonation product on the porosity and microcracks in concrete. Bouchaala et al. [4], reported that resonant ultrasonic nonlinear parameters have the feasibility of measuring concrete carbonation. Eiras et al. [5] used resonance ultrasonic spectroscopy technology to study the effect of concrete carbonation on the linear and nonlinear dynamic properties of cement-based materials. The linear and nonlinear dynamic properties can be used to deem material against carbonation in laboratory tests, whereas concrete has inherent nonlinearity due to its material composition. The nonlinear response of the concrete to the incident wave distorts the waveform when a single-frequency ultrasonic wave is transmitted into the concrete. Then, the higher harmonic waves will be generated in the transmitted wave. Kim et al. [6,7] investigated the feasibility of the second harmonic generation (SHG) technology of Rayleigh surface wave to characterize the carbonation of concrete. The amplitude of the active acoustic source of SHG technology is constant, while the percussive force of NURS needs to be control.

At present, health inspection of concrete structures is usually performed by manually carrying external ultrasonic probes. However, the use of large external probes requires extensive work preparation, which not only hinders the use of this technique for monitoring but also greatly limits the measurement area of concrete structures [8]. In contrast, embedding low-cost piezoelectric transducers in concrete structures allows for a more flexible configuration of the measurement network on the one hand, and long-term stable health measurements of the structure on the other. Such kinds of transducers have been successfully used for the monitoring of concrete hydration by deducing the evolution of Young’s modulus based on the flight time of the acoustic wave [9,10,11,12], the evolution of the concrete compressive strength at an early age [10,13], concrete cracking [8,14,15,16,17,18], water seepage [19], and mechanical properties of concrete as well as the acoustoelastic effect in compression [20,21,22,23], applying piezoelectric lead zirconate titanate (PZT) patches to the rebar by monitoring the conductance changes of the piezoelectric patches to study the effect of concrete carbonation on rebar corrosion [24,25]. In the current research of embedded transducers, it is found that cement-based and marble-based piezoelectric composite sensors have the feature of diminishing the distortion of signal and benefiting the energy transmission efficiency [26,27]. However, these large size transducers are not suitable for detecting P waves at close distances due to the carbonation of concrete developing from the surface. Qin et al. [12] and Chen et al. [28] provided two ideas for small size transducers in specimens with specific sizes. The signal transmitted by these transducers at an operating state with a high frequency and high energy carries less sensor nonlinearity than cement-based or marble-based transducers, whereas the influence of boundary reflection [6,29,30] on the signal should be considered in both time domain analysis and frequency domain analysis when the wave is propagating in small-size concrete. Otherwise, the parameters obtained from a small-size concrete are not suitable for the characterization of the large one due to the distance of boundary reflection.

In this paper, the ultrasonic transmission detection method was taken with an embedded transducer and a surface-mounted transducer. The embedded composite piezoelectric transducer (ECPT) which was composited with four materials was developed and used to detect the evolution of ultrasonic parameters in carbonated concrete by the SHG [31] technique of ultrasonic longitudinal waves to verify the effect of the concrete water–cement ratio on the relative nonlinear parameters of carbonation. This kind of ECPT had the advantage combined with the transducers used by Qin et al. [12] and Chen et al. [28], such as small size and electromagnetic shielding. Subsequently, the effect of the ambient temperature on the ultrasonic longitudinal detection parameters of carbonated concrete was also studied.

## 2. Materials and Methods

### 2.1. Specimens

In this experiment, concrete specimens (CI, CII, CIII) with different water–cement ratios were designed. The coarse aggregate used in the concrete was a continuous grade of natural gravel from 5 mm to 25 mm and the fine aggregate was natural river sand with a fineness modulus of 2.56. Table 1 shows the mixture design of the concrete specimens.

**Figure 1:**
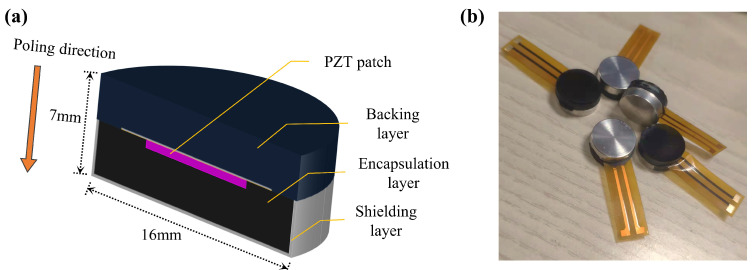
ECPT: (**a**) structure of ECPT; (**b**) the finished product of ECPT.

The structure of the embedded composite piezoelectric transducer (ECPT) is shown in Figure 1a. It consisted of a metal shielding layer, an encapsulation layer, a piezoelectric lead zirconate titanate (PZT) patch, and a composite backing layer. The model of the PZT was PSN-33 with a size of ϕ8mm×0.48mm, which was provided by the Haiying company. Table 2 shows the parameters of PZT, where $d_{33}$ is piezoelectric strain constant, *C* is capacitance, tanδ is dielectric dissipation factor, ft is resonance frequency, and Zr is impedance.

**Figure 2:**
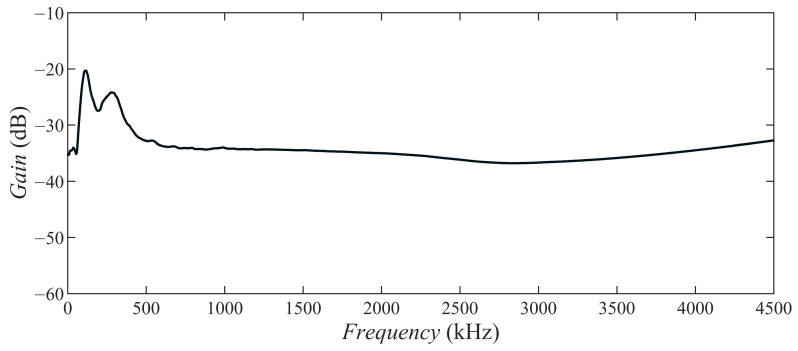
Response curves for ECPT.

This kind of composite increased the structural strength of the transducer and made it have good waterproofing and working performance. In addition, the ECPT could resist certain electromagnetic wave interference and suppress the interference of some noise to the signal during operation. The ECPT vibrated along the poling direction to generate mechanical waves when connected with the electric field. As shown in Figure 1b, the PZT patch is fastened to the printed circuit film in this transducer. This connection method ensured the working stability of the PZT patch and improved the convenience of the manufacturing process for the embedded composite transducer. The sensitivity of ECPT was verified with the face-to-face secondary calibration method (GB/T 19801-2005/ISO 12714:1999) [32,33,34]. The sensors were placed in the symmetry location to transmit and receive elastic waves from the solid medium surface—whereafter, the sensitivity of the sensor was calculated by the output signals. Figure 2 shows the sensitivity of ECPT.

**Figure 3:**
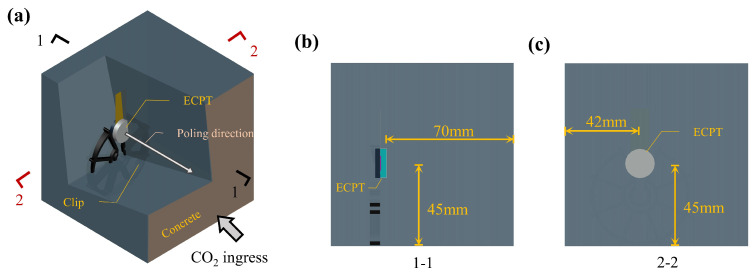
Arrangement of the embedded composite piezoelectric ultrasonic transducer: (**a**) partial cutaway perspective view of the specimen; (**b**) Section 1-1; (**c**) Section 2-2.

There were three specimens for each kind of concrete. Each size of the concrete specimen was 100 mm × 100 mm × 100 mm. As shown in Figure 3a, only one ECPT is embedded in the concrete specimen as a transmitter. On the surface in which CO${}_{2}$ was ingressed, a surface mounted transducer was fixed to obtain the nonlinear parameters of the ultrasonic radial propagation in carbonated concrete. The reason for this arrangement was that the received signal transmitted from inner concrete was experimentally found to be more stable than from the surface. In addition, this configuration could also be used in a large concrete structure. It was assumed that two transducers were embedded in one concrete and their poling direction pointed to the surface in which CO${}_{2}$ was ingressed, and the reflected P wave would be affected by the S wave [27].

As shown in Figure 3a, Section 1-1 is the plane perpendicular to the bottom surface of the concrete and along the central axis of the ECPT. Section 2-2 is perpendicular to the plane of the ECPT shielding layer on the bottom of the concrete in Figure 3a and the projection of ECPT on this plane is a circle. Figure 3b is Section 1-1 of the concrete in which the transducer is embedded. The central axis of the ECPT is 45 mm from the bottom surface of the specimen in Figure 3a. In addition, the concrete is up to 70 mm away from the surface of the shielding layer of the ECPT. Figure 3c is a Section 2-2 view of the concrete in which the transducer embedded. It can be found from the figure that the closest distance between the central axis of the ECPT and the surface of the concrete is 42 mm.

### 2.2. Accelerated Carbonation Experiment

Concrete specimens were cured in an environmental chamber with a temperature of 20 ± 3 °C and a relative humidity of 95%. After 28 days of curing, all surfaces of the specimens were sealed with paraffin wax except for the surface which CO${}_{2}$ ingressed. The concrete specimens were placed in the carbonation chamber for concrete carbonation experiments. In this case, the ambient temperature was 20 ${}^{\circ }$C, the CO${}_{2}$ concentration was 20%, and the relative humidity was 70%. The carbonation depth was measured with phenolphthalein in spare samples after 0, 7, and 21 days of carbonation.

### 2.3. Testing Methods

**Figure 4:**
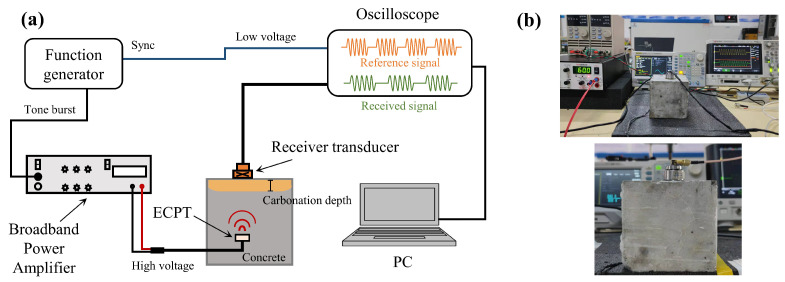
Experimental device: (**a**) connection of experimental instruments; (**b**) the photos of the experimental device.

The specimens were removed from the carbonation chamber after 3, 7, 14, and 21 days of concrete carbonation, respectively. In order to test the ultrasonic parameters of carbonated concrete under different temperature environments, the concrete was placed in an environment chamber at the temperature from −20 ${}^{\circ }$C to 40 ${}^{\circ }$C for 24 h with a temperature interval of 5 ${}^{\circ }$C, and the relative humidity was controlled at 70%. Figure 4 shows the experimental setup. A function waveform generator (DG 4202) generated 15-cycle, 1 MHz frequency, 10 peak-to-peak voltage sinusoidal waveform signal pulses at a burst interval of 3 ms. Then, the signal was amplified to a peak-to-peak voltage of 120 V by a broadband power amplifier (KROHN-HITE 7602M, Brockton, MA, USA), and the ultrasonic signal was transmitted by the ECPT in the concrete. It should be noted that the receiving transducer fixed to the concrete surface had the same central axis as the ECPT. The oscilloscope (Keysight InfiniiVision DSOX 3014T, Santa Rosa, CA, USA) was connected to the receiving transducer to acquire a time domain signal with a sampling length of 16,000 at a sampling frequency of 625 MSa/s, and the acquisition mode was the average acquisition of 2048 times. At the same time, a sync output line connected the function waveform generator to the oscilloscope to receive the reference waveform. The measurements of per specimen were repeated 10 times. Finally, received signal was subjected to post-processing such as denoising (wavelet transform), interception, windowing, and fast Fourier transform (FFT) by the computer.

**Figure 5:**
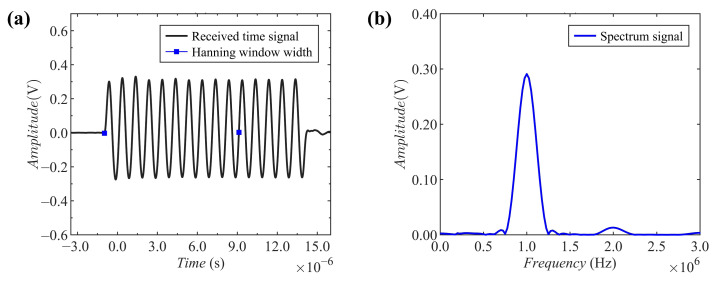
Time signal and spectrum for measurement: (**a**) received time domain signal; (**b**) spectrum signal after FFT ($A_{1}$: 1 MHz, $A_{2}$: 2 MHz).

The received time domain signal (the blue squares indicate the width of the Hann window) of carbonated concrete acquired by the oscilloscope is shown in Figure 5a. It should be noted that the time for the concrete boundary reflection wave to reach the receiving transducer measured in the experiment was around 28 μs. However, the pulse time length of the excitation wave was less than the time length required for the arrival of the reflected wave. In order to ensure maximum signal accuracy and avoid the influence of boundary reflection waves on the received time-domain signal, the first ten cycles of the time-domain signal were intercepted for windowing (Hanning) and subjected to FFT processing. The spectrum of the FFT processed signal is shown in Figure 5b, and the fundamental amplitude ($A_{1}$) and the second harmonic amplitude ($A_{2}$) can be observed from the figure. The products of the carbonation reaction filled the pores in concrete, resulting in an effect on the nonlinear response of concrete. Subsequently, the fundamental amplitude ($A_{1}$) and the harmonic amplitude ($A_{2}$) of the transmitted wave were changed by this effect. As such, the relative nonlinear parameter ($A_{2}/A_{1}^{2}$) could be assessed for the change in carbonated concrete.

## 3. Results and Discussion

The carbonation depth was measured with phenolphthalein in spare samples after 0, 7, and 21 days of carbonation. Table 3 shows the average carbonation depth (cd). The carbonation depth of concrete with different water cement ratio was different at the same carbonation time.

**Figure 6:**
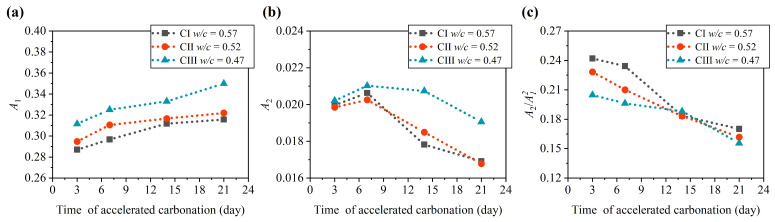
Variation of ultrasonic average parameters with carbonation time: (**a**) fundamental amplitude $A_{1}$; (**b**) second harmonic amplitude $A_{2}$; (**c**) nonlinear parameter $A_{2}/A_{1}^{2}$.

During the concrete carbonation progress, the existing pores and microcracks were deposited by the carbonation product $CaCO_{3}$ [5,35]. The molar volume of carbonated products was higher than that of hydrates, resulting in a decrease in the porosity of concrete and an increase in the density of concrete. These microstructural changes altered the ultrasonic parameters of the concrete. After 3, 7, 14, and 21 days of concrete carbonation, the concrete specimens were removed for ultrasonic measurement at 20 ${}^{\circ }$C. It can be observed from Figure 6 that the ultrasonic parameters change with the carbonation time of concrete. As shown in Figure 6b, the fundamental amplitude ($A_{1}$) of concrete increases with the concrete carbonation time. Meanwhile, the amplitude of the second harmonic ($A_{2}$) shows a decreasing trend in general. Hence, the relative nonlinear parameters ($A_{2}/A_{1}^{2}$) of concrete are reduced by the change of the fundamental amplitude ($A_{1}$) and the second harmonic amplitude ($A_{2}$). The variation trend of relative nonlinear parameters with the carbonation progress measured by the configuration at 20 ${}^{\circ }$C in this study is similar to that obtained by the second harmonic generation (SHG) technology of Rayleigh surface wave taken by Kim et al. [6,7]. The relative nonlinear parameters decrease with the carbonation progress. Although the water–cement ratios of CI, CII, and CIII are different, the variation of their ultrasonic parameters of carbonated concrete follows this trend.

**Figure 7:**
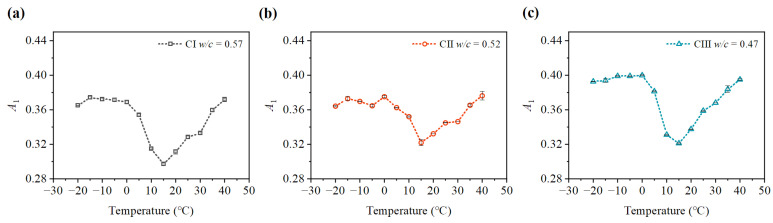
Variation of fundamental amplitude $A_{1}$ at different temperatures: (**a**) fundamental amplitude $A_{1}$ of CI; (**b**) fundamental amplitude $A_{1}$ of CII; (**c**) fundamental amplitude $A_{1}$ of CIII.

**Figure 8:**
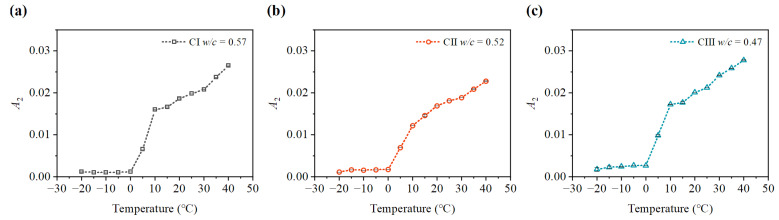
Variation of second harmonic amplitude $A_{2}$ at different temperatures. (**a**) second harmonic amplitude $A_{2}$ of CI; (**b**) second harmonic amplitude $A_{2}$ of CII; (**c**) second harmonic amplitude $A_{2}$ of CIII.

After 3, 7, 14, and 21 days of concrete carbonation, specimens were placed in an environment chamber at the temperature from −20 ${}^{\circ }$C to 40 ${}^{\circ }$C for 24 h with a temperature interval of 5 ${}^{\circ }$C, all with a relative humidity of 70%. Figure 7 and Figure 8 show the average fundamental amplitudes and second harmonic amplitudes of the ultrasonic longitudinal wave measured at different ambient temperatures after 14 days of concrete carbonation. The fundamental amplitudes and second harmonic amplitudes of the three kinds of concrete are different at various ambient temperatures. As shown in Figure 7, the fundamental amplitude is also affected by the increasing temperature from −20 ${}^{\circ }$C to 40 ${}^{\circ }$C. It can be seen that the fundamental amplitude measured at 15 ${}^{\circ }$C is the lowest. The fundamental amplitude ($A_{1}$) measured at 40 ${}^{\circ }$C increased significantly compared with that at 15 ${}^{\circ }$C. Then, the fundamental amplitude also rises in the temperature decreasing from 15 ${}^{\circ }$C to 0 ${}^{\circ }$C. This may be due to the trend of water freezing in concrete pores increasing the acoustic impedance of concrete. The fundamental amplitude ($A_{1}$) at −20 ${}^{\circ }$C does not change much from that at 0 ${}^{\circ }$C. It may be the reason that the water in the concrete pores at 0 ${}^{\circ }$C is in the state of a mixture of ice and water. Whereafter, the water in the concrete pores froze into ice at −20 ${}^{\circ }$C. As shown in Figure 8, the temperature reduction can not increase the second harmonic amplitudes ($A_{2}$). The second harmonic amplitudes ($A_{2}$) also rise after temperature increases ranging from 0 ${}^{\circ }$C to 40 ${}^{\circ }$C. Interestingly, the rising trend of the second harmonic amplitudes ($A_{2}$) is greater when the temperature ranges from 0 ${}^{\circ }$C to 10 ${}^{\circ }$C than when the temperature ranges from 10 ${}^{\circ }$C to 40 ${}^{\circ }$C. It can be seen clearly that the second harmonic amplitudes ($A_{2}$) of the three kinds of specimens decrease quickly with the temperature reduction. The amplitudes of the second harmonic decrease slightly at a temperature between −20 ${}^{\circ }$C and 0 ${}^{\circ }$C. Significantly, the amplitude of the second harmonic at low-temperature decreases by nearly 80% compared with that at 10 ${}^{\circ }$C.

**Figure 9:**
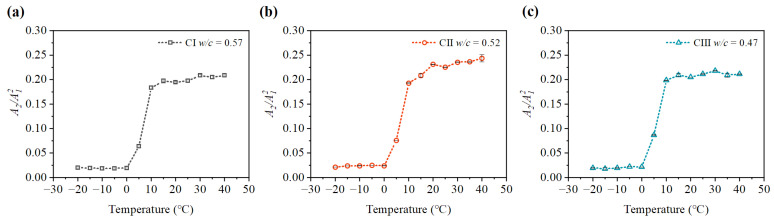
Variation of nonlinear parameter $A_{2}/A_{1}^{2}$ at different temperatures (day 3): (**a**) nonlinear parameter $A_{2}/A_{1}^{2}$ of CI; (**b**) nonlinear parameter $A_{2}/A_{1}^{2}$ of CII; (**c**) nonlinear parameter $A_{2}/A_{1}^{2}$ of CIII.

**Figure 10:**
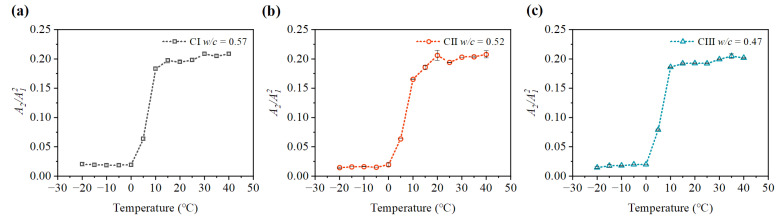
Variation of nonlinear parameter $A_{2}/A_{1}^{2}$ at different temperatures (day 7): (**a**) nonlinear parameter $A_{2}/A_{1}^{2}$ of CI; (**b**) nonlinear parameter $A_{2}/A_{1}^{2}$ of CII; (**c**) nonlinear parameter $A_{2}/A_{1}^{2}$ of CIII.

**Figure 11:**
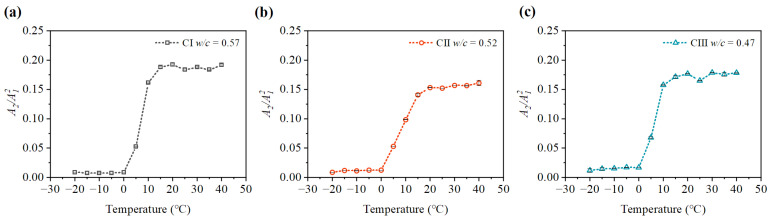
Variation of nonlinear parameter $A_{2}/A_{1}^{2}$ at different temperatures (day 14): (**a**) nonlinear parameter $A_{2}/A_{1}^{2}$ of CI; (**b**) nonlinear parameter $A_{2}/A_{1}^{2}$ of CII; (**c**) nonlinear parameter $A_{2}/A_{1}^{2}$ of CIII.

**Figure 12:**
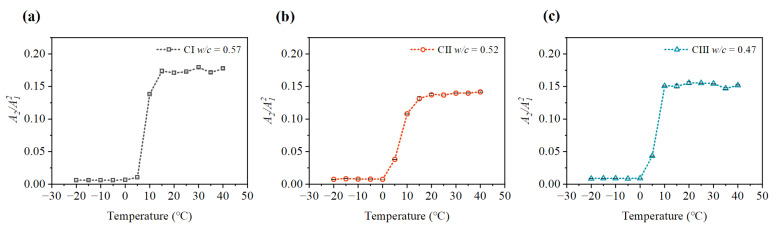
Variation of nonlinear parameter $A_{2}/A_{1}^{2}$ at different temperatures (day 21): (**a**) nonlinear parameter $A_{2}/A_{1}^{2}$ of CI; (**b**) nonlinear parameter $A_{2}/A_{1}^{2}$ of CII; (**c**) nonlinear parameter $A_{2}/A_{1}^{2}$ of CIII.

It can be seen from Figure 9, Figure 10, Figure 11 and Figure 12 that the relative nonlinear parameters ($A_{2}/A_{1}^{2}$) of carbonated concrete are changed by the influence of temperature during the carbonation time. The relative nonlinear parameters ($A_{2}/A_{1}^{2}$) measured at any temperature decrease with carbonation time. The relative nonlinear parameters of carbonated concrete measured at 0 ${}^{\circ }$C compared with that at 10 ${}^{\circ }$C decrease significantly (around 80 ∼ 90%), whereas the relative nonlinear parameters of carbonated concrete measured at 15 ${}^{\circ }$C to 40 ${}^{\circ }$C increase slightly. The relative nonlinear parameters also increase slightly when the temperature is at −20 ${}^{\circ }$C to 0 ${}^{\circ }$C, but it does not change too much. Table 4 shows the measured relative nonlinear parameters in different temperatures at the carbonation day 14.

**Figure 13:**
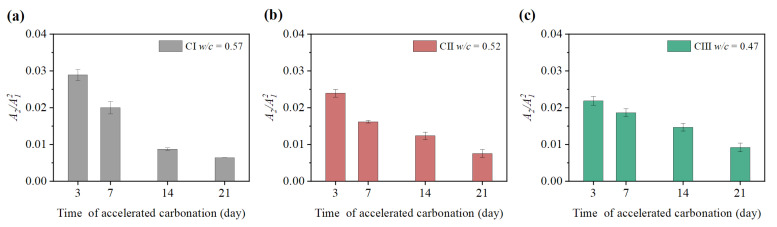
Variation of nonlinear parameter $A_{2}/A_{1}^{2}$ at 0 ${}^{\circ }$C: (**a**) nonlinear parameter $A_{2}/A_{1}^{2}$ of CI; (**b**) nonlinear parameter $A_{2}/A_{1}^{2}$ of CII; (**c**) nonlinear parameter $A_{2}/A_{1}^{2}$ of CIII.

**Figure 14:**
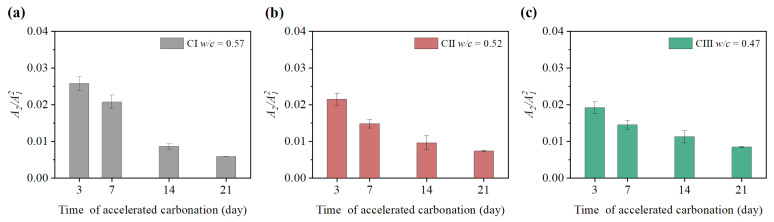
Variation of nonlinear parameter $A_{2}/A_{1}^{2}$ at −20 ${}^{\circ }$C: (**a**) nonlinear parameter $A_{2}/A_{1}^{2}$ of CI; (**b**) nonlinear parameter $A_{2}/A_{1}^{2}$ of CII; (**c**) nonlinear parameter $A_{2}/A_{1}^{2}$ of CIII.

As shown in Figure 9, Figure 10, Figure 11 and Figure 12, the values of the relative nonlinear parameters are more minor at 0 ${}^{\circ }$C than at 10 ${}^{\circ }$C, whereas the measured nonlinear parameters for the three concrete specimens change obviously with carbonation time in low-temperature environments as shown in Figure 13 and Figure 14. In the same carbonation environment, the carbonation rate of concrete with high water–cement ratios is faster than that with low water–cement ratios [36]. The data in Table 3 follow this trend and can also be reflected from the changes of the relative nonlinear parameters in Figure 13 and Figure 14. In addition, the variational degree of nonlinear parameter is greater for CI (w/c = 0.57) than for CII (w/c = 0.52) and CIII (w/c = 0.47). It means that the detection method in this study is suitable for the assessment of carbonated concrete in low-temperature environments as well.

## 4. Conclusions

In this paper, the longitudinal nonlinear ultrasonic parameters of carbonated concrete were detected using an embedded composite piezoelectric transducer (ECPT) as the transmitter, and the effect of temperature on the nonlinear parameters of carbonated concrete is investigated. Conclusions are drawn from the experimental results as follows:

- Though only one ECPT was embedded in the concrete specimen as a transmitter, the nonlinear parameters of the ultrasonic radial propagation in carbonated concrete could also be obtained by the surface-mounted transducer fixed on the surface in which CO${}_{2}$ was ingressed. This configuration can detect concrete carbonation with a monitoring network for a long time without damage when the surface-mounted transducer is permanently fixed on the structural surface.
- The relative nonlinear parameter of carbonated concrete can be affected by the water–cement ratio of the concrete. There is a more significant variation of the nonlinear parameters in the same carbonation time for the specimens with a high water–cement ratio than that with a low one. It indicates that the change of relative nonlinear parameters is closely related to the concrete carbonation depth.
- At the same ambient relative humidity (RH = 0.7), the fundamental amplitudes ($A_{1}$) and the second harmonic amplitudes ($A_{2}$) are increased by the increasing temperature from 15 ${}^{\circ }$C to 40 ${}^{\circ }$C, and the relative nonlinear parameters ($A_{2}/A_{1}^{2}$) between the two temperatures are also increased slightly. The fundamental amplitude of carbonated concrete at temperatures below 15 ${}^{\circ }$C increases dramatically, while the second harmonic amplitude decreases sharply below 0 ${}^{\circ }$C compared with that at 10 ${}^{\circ }$C (fell around 80%). The relative nonlinear parameters of carbonated concrete measured at 0 ${}^{\circ }$C compared with that at 10 ${}^{\circ }$C decreases significantly (around 80∼90%). The relative nonlinear parameters also increase slightly when the temperature is at −20 ${}^{\circ }$C to 0 ${}^{\circ }$C, but it does not change too much. As such, the relative nonlinear parameters in low temperatures below 0 ${}^{\circ }$C are much smaller than at temperatures above 10 ${}^{\circ }$C. It may be related to the water condensation in the concrete. In addition, the detection method in this study is also appropriate for the measurement of carbonated concrete in low-temperature environments, since the measured nonlinear parameters for the three concrete specimens change obviously with carbonation time.
- Considering the effect of temperature variations on the relative parameters of carbonated concrete, temperature compensation should be performed for the relative nonlinear parameters when continuously monitoring the concrete carbonation process.

It can be seen from the results that the relative nonlinear parameters change significantly at the temperature from 0 ${}^{\circ }$C to 10 ${}^{\circ }$C. The variation of the relative nonlinear parameter in this temperature interval needs to be studied further. Moreover, future studies could investigate the quantitative association between the relative nonlinear parameters and concrete carbonation depth for different kinds of concrete. In addition, the other factors in the measurements for carbonated concrete nonlinear parameters will be investigated in more detail.

## Figures and Tables

**Table 1:** Mixture ratio of the concrete specimen.

Category	CI	CII	CIII
OPC ($\mathrm{kg}/m^{3}$)	342	375	415
Fine aggregate ($\mathrm{kg}/m^{3}$)	708	695	680
Coarse aggregate ($\mathrm{kg}/m^{3}$)	1155	1135	1110
Water ($\mathrm{kg}/m^{3}$)	195	195	195

**Table 2:** The parameters of PZT.

Model	$d_{33}$ ($\mathrm{pC}\cdot N^{-1}$)	*C* (pF)	tanδ (%)	ft (MHz)	Zr (Ω)
PSN-33	377	1167	1.77	4.03	5.45

**Table 3:** The average carbonation depth.

	0 Day–cd (mm)	7 Day–cd (mm)	21 Day–cd (mm)
CI w/c = 0.57	0.51	6.41	11.83
CII w/c = 0.52	0.32	5.17	9.51
CIII w/c = 0.47	0.11	3.65	7.12

**Table 4:** The measured average relative nonlinear parameters.

Temperature (${}^{\circ }$C)	−20	−15	−10	−5	0	5	10	15	20	25	30	35	40
CI w/c = 0.57	0.00867	0.00842	0.00752	0.00843	0.00951	0.05216	0.16068	0.18743	0.19172	0.18288	0.18733	0.18284	0.19091
CII w/c = 0.52	0.01052	0.01169	0.01128	0.01135	0.01236	0.05288	0.0984	0.14073	0.15304	0.15215	0.15699	0.15659	0.16099
CIII w/c = 0.47	0.01362	0.01453	0.01512	0.01587	0.01564	0.06761	0.15755	0.17139	0.17661	0.16495	0.17859	0.17563	0.17809

## Data Availability

Not applicable.
